# A Translational Model to Improve Early Detection of Epithelial Ovarian Cancers

**DOI:** 10.3389/fonc.2022.786154

**Published:** 2022-04-21

**Authors:** Allison Gockley, Konrad Pagacz, Stephen Fiascone, Konrad Stawiski, Nicole Holub, Kathleen Hasselblatt, Daniel W. Cramer, Wojciech Fendler, Dipanjan Chowdhury, Kevin M. Elias

**Affiliations:** ^1^ Division of Gynecologic Oncology, Department of Obstetrics, Gynecology and Reproductive Biology, Brigham and Women’s Hospital, Boston, MA, United States; ^2^ Harvard Medical School, Boston, MA, United States; ^3^ Studies in Molecular Medicine, Medical University of Warsaw, Warsaw, Poland; ^4^ Department of Biostatistics and Translational Medicine, Medical University of Lodz, Lodz, Poland; ^5^ Obstetrics and Gynecology Epidemiology Center, Department of Obstetrics and Gynecology and Reproductive Biology, Brigham and Women’s Hospital, Boston, MA, United States; ^6^ Department of Radiation Oncology, Dana-Farber Cancer Institute, Boston, MA, United States

**Keywords:** ovarian cancer, screening, microRNA, xenograft, neural networks

## Abstract

Neural network analyses of circulating miRNAs have shown potential as non-invasive screening tests for ovarian cancer. A clinically useful test would detect occult disease when complete cytoreduction is most feasible. Here we used murine xenografts to sensitize a neural network model to detect low volume disease and applied the model to sera from 75 early-stage ovarian cancer cases age-matched to 200 benign adnexal masses or healthy controls. The 14-miRNA model efficiently discriminated tumor bearing animals from controls with 100% sensitivity down to tumor inoculums of 50,000 cells. Among early-stage patient samples, the model performed well with 73% sensitivity at 91% specificity. Applied to a population with 1% disease prevalence, we hypothesize the model would detect most early-stage ovarian cancers while maintaining a negative predictive value of 99.97% (95% CI 99.95%-99.98%). Overall, this supports the concept that miRNAs may be useful as screening markers for early-stage disease.

## Introduction

In the United States, over 22,000 women annually are diagnosed with ovarian cancer and over 14,000 die of their disease (1). Currently, most ovarian cancers are detected at an advanced stage, where 5-year survival rates average 25-30% (2). In contrast, stage I ovarian cancers have 5-year survival rates in excess of 90% (3). Detection of more ovarian cancers at an earlier stage would therefore be expected to improve long-term survival (4). Prior efforts to screen for ovarian cancer have included 2 large randomized controlled trials utilizing the serum biomarker CA-125 and pelvic ultrasound; unfortunately, both of these trials failed to show a survival benefit (5–7). This is likely because neither CA-125 nor pelvic ultrasound is sufficiently sensitive to detect low volume disease (8).

In a prior report, we described a neural network model which used 14 serum microRNAs (miRNAs) to predict the presence of ovarian cancer (9). miRNAs are small non-coding RNAs (18-24 nucleotides) which modify gene expression through post-transcriptional regulation. A growing body of evidence suggests that miRNAs are aberrantly expressed in ovarian cancer (10–19). Other groups have similarly suggested circulating miRNAs might be useful as non-invasive diagnostic or prognostic tools (20, 21). However, the applicability of these miRNA models to small tumor volume and early tumor stage is uncertain. Both tumor volume and the relative isolation of the human ovaries from circulation are challenges for ovarian cancer early detection. This reflects the relative disconnect between stage and tumor volume in ovarian cancer staging, e.g., a palpable 30 cm tumor may be Stage I while an occult 3 mm peritoneal implant may impart Stage III. This poses problems when creating animal models to test early detection biomarkers. The mouse ovary contains a bursa surrounding the tubo-ovarian interface, whereas the human tubo-ovarian interface is continuous with the peritoneal cavity. Injection of small volume disease in the mouse peritoneum accurately reflects some aspects of early onset human disease, i.e. metastasis early in disease development, but it cannot account for the immunologic sequestration of very early tumors within the ovary or fallopian tube. Similarly, intra-ovarian injection of tumor cells fails to account for the exposure of the human ovary to the peritoneal cavity.

Here, we use a xenograft model to improve our early detection signature with respect to both challenges. First, we improve our existing ovarian cancer prediction model by using an animal model to sensitize the neural network to low volume disease. Next, after recalibrating the model to focus on low tumor volume, we show that these same miRNAs can be used to construct a diagnostic model that performs well for identifying patients with early-stage disease.

## Materials and Methods

### Ethics Statement

All clinical investigations were conducted according to Declaration of Helsinki principles. Sera from cases and benign masses were collected under the Pelvic Mass Protocol (Brigham and Women’s Hospital Institutional Review Board Protocol 2000-P-001678) and the New England Case Control Study (Dana-Farber Cancer Institute Institutional Review Board Protocol 05–060) (22). Samples for the present study were collected between 2001 and 2016. All subjects were enrolled after signing written informed consent.

### Animals

Animal experiments were conducted in accordance with the Dana-Farber Cancer Center Animal Resource Facility Ethics Guidelines (IACUC protocol 13-043). All animals were 8-week old female NOD-SCID-Gamma (NSG) immunocompromised mice obtained from the Jackson Laboratory (Bar Harbor, Maine).

### Development of Engrafted Murine Models

Three luciferized human high-grade serous ovarian cancer (HGSOC) cell lines (COV362, Kuramochi, and OVSAHO) were grown in DMEM-F12 medium with 5% fetal bovine serum and 1% penicillin/streptomycin at 37° Celsius with 5% CO2. Prior to injection, lines were tested for mycoplasma infection *via* the Mouse/Rat Comprehensive Clear Panel (Charles River Research Animal Diagnostic Services, Wilmington, Massachusetts). Cell line identities were confirmed by short tandem repeat loci testing (ATCC, Manassas, Virginia). Ten NOD-SCID-Gamma (NSG) immunocompromised mice were obtained from the Jackson Laboratory (Bar Harbor, Maine) and divided in 4 groups for injection. Three mice received COV362 cells, 3 mice received Kuramochi cells and 3 mice received OVSAHO cells injected with the technique as described below. These mice were injected with a total of 5 million tumor cells. One mouse served as a control mouse in each cage and did not receive tumor cells. All animals were routinely monitored for signs of poor condition and euthanized according to animal staff recommendations.

On day 1 of the experiment, the control mouse underwent peritoneal injection with 200uL of a 1:1 mixture of Matrigel^®^ Matrix (Corning) and DMEM F12 media. The other mice were injected with 200uL of a 1:1 mixture of Matrigel and 5 million HGSOC cells. On day 8 all mice underwent injection with 200uL of 30mg/mL D-luciferin and images were acquired starting 10 minutes after luciferin injection. Immunofluorescence imaging data was collected at this time point to verify tumor engraftment. Mice were then euthanized. Tissue samples were harvested *via* micro-dissection techniques. Tissues were then plated with Beetle Luciferin (15.0 mg/mL) at a 1:100 dilution with media and then imaged with a plate reader for 2 minutes to identify microscopic tumors. Additional tumor-bearing tissues were then embedded in optimal cutting temperature compound (OCT) and snap frozen for histologic analysis. These OCT-embedded tissues were then cut *via* a cryotome and then placed on slides for hematoxylin and eosin staining and immunohistochemistry for PAX8 antibodies to further confirm tumor engraftment.

### Identification of Serum miRNA From Engrafted Human HGSOC in a Low-Volume Murine Model

After confirming that the HGSOC cell lines engrafted in mice, the same human HGSOC cell lines (COV362, Kuramochi, and OVSAHO) were used to model low-volume disease and collect murine serum for analysis. The experiment was repeated twice for a total of 10 mice in each treatment group (40 mice total). Mice underwent a baseline submandibular blood collection prior to tumor inoculation. On day 1 of the experiment, 5 control mice per experiment underwent intraperitoneal injection with 200uL of a 1:1 mixture of Matrigel^®^ Matrix (Corning) and DMEM F12 media. In parallel, 5 experimental mice per group per experiment were injected with 200uL of a 1:1 mixture of Matrigel and 500,000 cells of COV362 cells, Kuramochi cells, or OVASHO cells. On day 5, the mice underwent another submandibular blood collection and injection with 200uL of 30mg/mL D-luciferin. Images were acquired starting 10 minutes after luciferin injection. The immunofluorescence data was collected at this time point to verify tumor engraftment. On day 28 another round of immunofluorescent imaging was completed identically to the day 5 procedure and the mice underwent another submandibular blood collection. Mice were monitored for tumor growth and general health and then euthanized on day 28, unless moribund earlier. All animals were routinely monitored for signs of poor condition and euthanized per animal staff recommendations.

Serum from each collection time point was aliquoted into a 96-well PCR plates and subsequently all serum was analyzed using oligonucleotide probes to miRNAs using the Fireplex platform (Abcam, Cambridge, MA). This assay involves extracting miRNAs from crude biofluid followed by hybridization to target-specific probes embedded in barcoded hydrogel particles. The labeled miRNAs then undergo one-step RT-PCR with a biotinylated primer. PCR products are then re-hybridized to the original particles and are incubated with a reporter for detection. A flow cytometer is then used to detect the particles. Signals generated are proportional to the average amount of fluorescent target bound to the particles. The miRNAs investigated in this experiment were based on the 14 predictive miRNAs and 9 miRNAs with relatively stable serum levels (“normalizers”) identified previously by Elias, et al. (9). The predictive miRNAs were: miR-23b-3p, miR-29a-3p, miR-32-5p, miR-92a-3p, miR-150-5p, miR-200a-3p, miR-200c-3p, miR-203a, miR-320c, miR-320d, miR-335-5p, miR-450b-5p, miR-1246 and miR-1307-5p. Controls included miRNAs from other species to correct for background fluorescence, spike-in positive controls, and no specimen blanks.

The experimented was then repeated with a distinct HGSOC cell line, OVCAR8. A total of 30 mice were used (15 controls and 15 xenografts). The xenograft mice were divided into 3 groups: 5 mice received an injection of 50,000 cells, 5 mice received an injection of 500,000 and 5 animals were injected with 5 million cells. Mice were randomized among cages using a random number generator (www.random.org). Similar to the prior experiments, baseline, day 5 and day 28 submandibular blood samples were collected, and bioluminescent imaging was obtained. Serum samples in triplicate were randomized onto plate locations for analysis using a random sequence generator (www.random.org), with technical replicates randomized from another. Investigators were blinded to the treatment allocation during the interpretation of the bioluminescent images and the analysis of the serum samples. Experimental group identities were assigned using a coded key once the analysis was complete to construct the receiver operating characteristic curves.

### Multilayer Perceptron Neural Network Design

To design a model to distinguish between murine control and tumor-bearing serum samples, a multilayer perceptron model (MLP) was employed. This is an artificial neural network consisting of, in our case, 3 layers of nodes: an input layer, a hidden layer and an output layer. Serum samples were allocated to training (used for model development) and testing (used to evaluate training, overfitting and cut-off calibration) sets. The testing and training sets were derived from the experiment utilizing 500,000 cells of COV362, Kuramochi and OVSAHO lines. In total, 40 animals were used: 10 controls and 10 inoculated with cells of each of the three cell lines, with serum samples processed in triplicates. The serum from this experiment was randomly assigned using a random number generator (www.random.org): 25% to the test set and 75% to the training set. An independent validation set then consisted of the serum data from the OVCAR8 experiment as described above (30 mice: 15 controls, 15 inoculated with tumor cells). Expression of 9 normalizer miRNAs, previously described in Elias et al., was assayed simultaneously to the 14 predictive miRNAs (9). Analysis was then conducted on 15,000 models with the top 15 models evaluated manually. The previously defined 14 miRNAs were normalized to the top two most stable miRNAs in the FirePlex assay (miR-222 and miR-181a) (23). The MLP model was created with an empirically optimized number of neurons in the hidden layer and empirically selected linking functions. Once created, the network was refined by removing miRNAs starting with those classified as least useful for network performance in terms of overall error of classification. This allowed for an empirically optimized number of neurons in the hidden layer and empirically selected linking functions. This process was repeated until no further miRNAs could be removed. The final model included: miR-150, miR-200a, miR-200c, miR-203a, miR-320d, miR-335 and miR-405b.

For the human samples, the neural networks were constructed similarly. Samples were randomized 3:1:1 to training, testing, and validation sets using a random number generator (www.random.org). We built over 100000 neural networks based on the 14 signature miRNAs and retained the best one in terms of performance in properly assigning cases to classes in the test set. The networks were built in a semi-automated way. Their structure was of a multilayer perceptron with a number of neurons in the hidden layer iteratively optimized from (n variables)/3 to (n variables)*1.5 to avoid overfitting. Admissible linking functions between the neuron layers were linear, logistic, hyperbolic tangential, and exponential. Neuron weights were calculated using the BFGS (Broyden-Fletcher-Goldfarb-Shanno) algorithm and the network was trained in each epoch using an error back-propagation algorithm to optimize weights in each pass. The code files for the neural networks used for all analyses are available in the **Supplementary Materials**.

### Human Samples

Serum samples were collected fresh in 13 × 75 mm BD Vacutainer Plus Plastic Serum tubes (BD Life Sciences, Franklin Lakes, NJ) with spray-coated silica. Samples were allowed to clot 1 hour at room temperature before processing, then spun down by centrifugation at 1300 x g x 10 min, aliquoted into 1.5 ml vials and stored at – 80 C. Samples were thawed and aliquoted for the current study and then refrozen. There was no overlap of subjects between the current study and our prior report (9). For each study subject, pathology reports were re-reviewed to confirm clinical information and to accurately stage patients according to the most recent staging guidelines from the International Federation of Gynecology and Obstetrics (FIGO) (24).

### Next Generation Sequencing

Sample preparation, library construction, and miRNA sequencing were performed by Qiagen, Inc. (Frederick, MD). 500 μl of human serum from each sample were analyzed in duplicate. Total RNA from each serum sample was isolated using the manufacturer’s protocol optimized for serum. The quality of the isolated RNA was checked using qPCR. Total RNA was converted into microRNA NGS libraries using the NEBNEXT library generation kit (New England Biolabs Inc., Ipswich, MA). Adaptors were ligated to its 3’ and 5’ ends and converted into cDNA. cDNA was pre-amplified with specific primers containing sample-specific indices. After 18 cycles of pre-PCR the libraries were purified on QiaQuick columns and the insert efficiency evaluated by a Bioanalyzer 2100 instrument on a high sensitivity DNA chip (Agilent Inc., Lexington, MA). The microRNA cDNA libraries were size fractionated on a LabChip XT (PerkinElmer Waltham, MA) and a band representing adaptors and a 15–40 bp insert excised. Samples were then quantified using qPCR and concentration standards. Based on the quality of the inserts and the concentration measurements, the libraries were pooled in equimolar concentrations, quantified again with qPCR, and the optimal concentration of the library pool used to generate the clusters on the surface of a flowcell before sequencing using v3 sequencing methodology according to the manufacturer instructions (Illumina Inc., Dedham, MA). Samples were sequenced on the Illumina NextSeq 500 system (Illumina Inc., Dedham, MA) using a single-end read length of 50 nucleotides at an average of 10 million reads per sample. On the raw reads, adapter trimming and filtration was performed (using fastp tool https://github.com/OpenGene/fastp). Preprocessed reads were further mapped to the miRbase (version 22, http://www.mirbase.org/) using bowtie mapper (version 1.2.3). Feature counts were converted to tags per million (TPM) without correction for the library size.

## Results

### Human miRNAs Associated With Ovarian Cancer Are Detectable in the Serum of Murine Ovarian Cancer Xenografts

**Figure 1:**
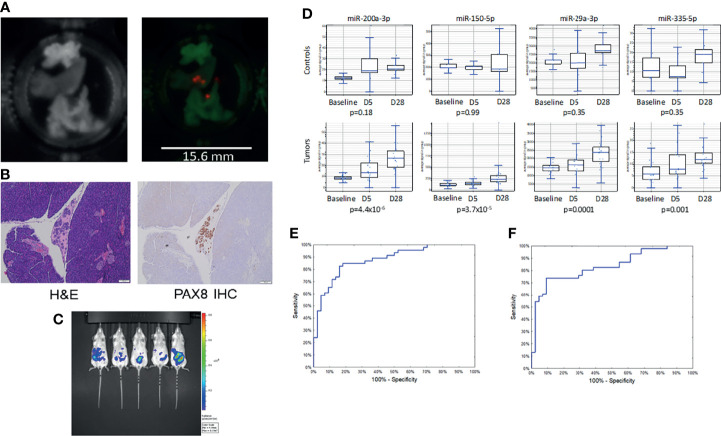
Low volume disease model of ovarian cancer growth. **(A)** Photograph (l) and bioluminescent images (r) of explanted organs showing sub-millimeter tumor growth at 1 week post-implantation. **(B)** Micrographs of miliary lesions as seen by hematoxylin and eosin (l) and immunohistochemical staining for the serous carcinoma marker PAX8 (r). **(C)** Bioluminescent images of mice taken 28 days post-injection with 500,000 tumor cells. **(D)** Serial miRNA levels among control (n = 5) vs. tumor bearing (n = 15) mice. p-values for trend from baseline to 28 days. **(E)** Receiver operating characteristic curve for the neural network using the full 14 miRNA signature (AUC = 0.88) or **(F)** a reduced set of 7 miRNAs (AUC = 0.85).

In our prior report, we presented a panel of 14 miRNAs which could distinguish women with ovarian cancer from those with benign pelvic masses or healthy controls (9). To test the feasibility of measuring these same miRNAs in the circulation of xenografts, we inoculated immunocompromised mice with intraperitoneal tumors using three human ovarian cancer cell lines (Kuramochi, COV362, and OVSAHO) known to generate small tumor implants (25). Tumors were identified by microdissection and *ex vivo* bioluminescent imaging with a high sensitivity charge-coupled device (CCD) camera. This technique allowed identification of tumors less than 1 mm in diameter, which could be identified in all 3 cell lines (**Figure 1A**). The small volume implants were then confirmed by histologic examination and immunohistochemical staining using the Mullerian carcinoma marker PAX8 (**Figure 1B**). Mice were inoculated with 500,000 cells per mouse or PBS control (n=10 per cell line or control), and then assessed serially by bioluminescent imaging over 28 days (**Figure 1C**). Whereas serum miRNA levels remained stable in control animals, tumor growth was associated with a progressive increase in serum levels of several tumor-associated miRNAs (**Figure 1D**).

The sera from the mice were then randomly divided into training and test sets. Following our previously described method, a neural network in the form of a multilayer perceptron (MLP) was trained using the 14 miRNAs. The model had good performance, with an AUC of 0.88 (95%CI 0.81-0.95; **Figure 1E**). There was little evidence of overfitting with overall accuracy being similar for the training set and the test set. In the training and test sets, sensitivity, specificity and accuracy equaled: 82.6%/84.1%/83.3% and 76.9%/93.8%/86.2%, respectively. To refine the model, we performed a global sensitivity analysis, removing the miRNAs that contributed the least to model performance. This reduced set consisted of only 7 miRNAs: hsa-miR-150, hsa-miR-200a, hsa-miR-200c, hsa-miR-203a, hsa-miR-335, hsa-miR-450b, and hsa-miR-320d. The simpler model had a similar AUC of 0.85 (95%CI 0.77-0.93; **Figure 1F**) but relied on fewer markers. For the training and testing sets the sensitivity, specificity, and overall accuracy of the reduced model were 73.9%/86.4%/80.0% and 76.9%/87.5%/82.8%, respectively. The row measurements in duplicates and group assignments are available in **Supplementary Data Sheet 1** (experiments 1 and 2).

### miRNAs Identify Mice Bearing Unrelated Tumors Regardless of Tumor Volume

**Figure 2:**
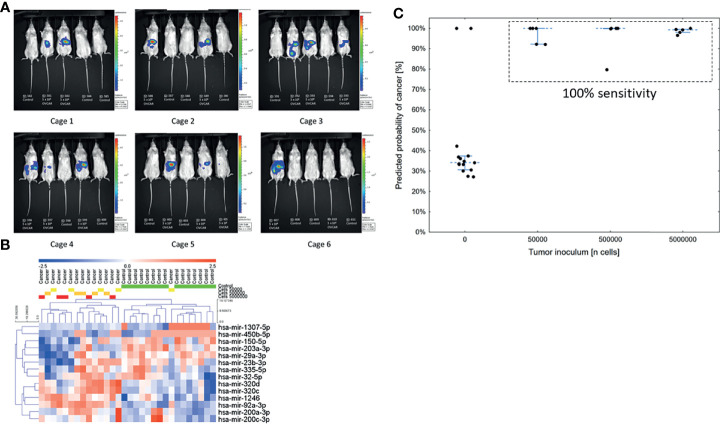
Validation of the low volume disease model in an independent cell line across tumor volumes (n = 30). **(A)** Bioluminescent images of mice taken 28 days post-injection. Groups were randomized among the cages. **(B)** Unsupervised hierarchical clustering using only the 14 miRNAs previously reported in the serum neural network. **(C)** Predicted probability of cancer in the serum samples at 28 days using a neural network model. The cut-off for a positive test was set at 50%.

Notably, Kuramochi, COV362, and OVSAHO all bear mutations in *BRCA1* or *BRCA2.* To validate the performance of the model in a non-*BRCA* mutated cell line, as well as to test the sensitivity of the model to low volume disease, we examined murine xenografts implanted with a fourth ovarian cancer cell line, OVCAR8 (n=30), not used to train the previous models and known to be wild-type for *BRCA1* and *BRCA2*. Mice received a logarithmic dose range of tumor cells: 50,000, 500,000, or 5 million cells injected intraperitoneally. Tumor injection volumes or PBS placebo injection assignments were randomized among the cages (**Figure 2A**). Bioluminescent imaging and serum miRNA measurements were performed blinded to the inoculum groups. The serum miRNA profiles distinguished tumor-bearing mice from controls, but profiles did not cluster by tumor inoculum (**Figure 2B**). This suggests that over the course of the study serum miRNA levels reached a steady-state. Consistent with this hypothesis, the 7-miRNA neural network produced 100% sensitivity at 86.7% specificity for discriminating tumor-bearing mice from control mice (overall accuracy of 93.3%) (**Figure 2C**). Although there were two false-positives, the model correctly identified all tumor-bearing mice regardless of the original tumor inoculum. The row values of the measurements for this part of the analysis are available in **Supplementary Data Sheet 2** (experiment 3).

### Serum miRNAs Can Distinguish Human Cases of Early-Stage Ovarian Cancers From Benign Masses or Healthy Controls

Having shown experimentally that the serum miRNA neural network is relatively insensitive to tumor volume, we next assessed the performance of the model among specifically early-stage cases (FIGO stage I-IIIA2). Small RNA sequencing was used to generate serum miRNA profiles of 275 study subjects comprising 75 cases, 100 benign adnexal masses, and 100 healthy controls (**Table 1**). Cases, benign masses, and controls were matched for age (mean 57, 55, and 55 years, respectively). The mean CA-125 among cases was 401 IU/ml (range 2-3725), with 19 cases (25.3%) having a CA-125 < 35 IU/ml, which is the upper limit of normal in a post-menopausal woman, and 42 cases (56%) having a CA-125 < 200 IU/ml, which is the upper limit of normal in a pre-menopausal woman. Among the cases, 44 (58.7%) were high-grade histologies.

**Table 1:** Early-stage case-control cohort.

Characteristic	N=275
Age, y, median, (range)	55 (24.0 – 84.0)
CA-125 (IU/ml), median (range)	28 (2 - 3725)
Diagnosis, n (%)	
Healthy Control Benign mass Endometrioma Serous cystadenoma Cancer	100 (36.4) 100 (36.4) 25 (9.1) 75 (27.3) 75 (27.3)
Cancer Histology, n (%)	
Serous Endometrioid Clear Cell Carcinosarcoma Transitional Cell Mucinous	18 (24.0)
	29 (38.7)
	23 (30.7)
	2 (2.7)
	2 (2.7)
	1 (1.3)
Cancer FIGO^A^ Stage, n (%)	
IA IB IC IIA IIB IIIA1 IIIA2	25 (33.3)
	3 (4.0)
	22 (29.3)
	14 (18.7)
	8 (10.7)
	2 (2.7)
	1 (1.3)
Cancer Grade, n (%)	
Borderline 1 2 3	2 (2.7)
	20 (26.7)
	9 (12.0)
	44 (58.7)
Genetic testing among cases, n (%)	
n/a^B^ negative BRCA1 BRCA2 RAD51C	50 (66.7)
	21 (28)
	1 (1.3)
	2 (2.7)
	1 (1.3)

^A^FIGO Stage – per International Federation of Gynecology and Obstetrics 2014 staging guidelines. ^B^n/a – not available

**Figure 3:**
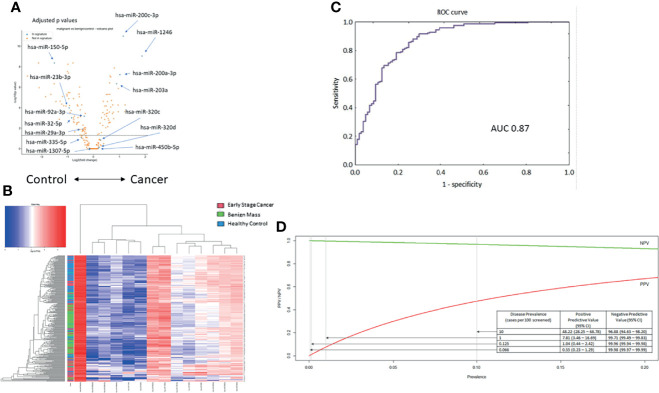
Testing the 14-miRNA signature in the early-stage ovarian cancer cohort (n = 275). **(A)** Volcano plot for all miRNAs in the samples. Selected miRNAs from the neural network are highlighted. Values adjusted for multiple testing. **(B)** Unsupervised hierarchical clustering using only the 14 miRNAs previously reported in the serum neural network. **(C)** Receiver operating characteristic curve for the neural network to distinguish early-stage cancer from benign masses or healthy controls (AUC = 0.87). **(D)** Modeling the performance of the neural network classifier by disease prevalence among hypothetical populations.

In univariate analysis of the miRNA profiles, the components of the miRNA serum signature were well-represented (**Figure 3A**). However, as we have described in prior reports, univariate measures of serum miRNA expression were insufficient to classify samples based on hierarchical clustering (**Figure 3B**). The samples were then divided into training, testing, and validation sets in proportions of 3:1:1 for calibration of the animal model to the human samples. For the validation set, the model had an AUC of 0.87 (95%CI 0.84-0.94; **Figure 3C**). Using a predicted probability for cancer of 50% as the cut-off for a positive test result, the model had 73% sensitivity at 91% specificity for distinguishing early-stage ovarian cancers from benign adnexal masses or healthy controls.

Notably, ovarian cancer has a low prevalence in an unselected general population. Therefore, we modeled how the performance of the miRNA classifier would vary based on disease prevalence. Based on these results, we hypothesize that application of the test would require a population with an underlying disease prevalence of at least 1% (**Figure 3D**). Among such a population, a positive test result would indicate an almost 8-fold increased risk of ovarian cancer, which could then prompt further evaluation, while maintaining a negative predictive value of 99.71%.

## Discussion

In this study, we investigated the effects of two variables which might impact a neural network based on serum miRNAs, namely disease volume and disease stage. In our animal model, the miRNAs were able to detect tumors below the threshold of bioluminescent imaging and at tumor volumes not visible to the human eye. The predictive capacity for the model translated well to a large cohort of early-stage human samples, covering a variety of histologies and clinical presentations.

miRNAs have been studied extensively in both ovarian cancer as well as other solid tumor disease sites. Due to the stability of miRNAs in serum, miRNAs are particularly attractive as diagnostic biomarkers for early-stage disease. For example, Tang, et al. compared miRNAs from 36 early-stage gastric cancer patients to those from 12 healthy individuals and described how a subset of the miRNAs could serve as potential non-invasive biomarkers for early diagnosis of gastric cancer with AUC of up to 0.786 (26). Similarly, in prostate cancer, Fredsoe, et al. published a study utilizing 753 patients including those with benign prostatic hyperplasia, localized prostate cancer, advanced prostate cancer, and non-cancer controls. The authors were able to build a model based on 4 miRNAs in combination with clinical factors that outperformed PSA alone with AUC 0.84 (27). Among gynecologic malignancies, the role of miRNAs has also been studied in endometrial adenocarcinoma. Wang et al. recently published a machine-learning model using TCGA atlas data which identified 9 miRNAs as diagnostic markers with overall correct rates of distinguishing benign from tumor tissue of > 95% (28). 5 miRNAs were then used to construct a prognostic model which identified patients at high risk of mortality more accurately than clinical stage (28). Non-coding RNAs appear to play an essential role in endometrial cancer pathogenesis, and non-coding RNAs may prove to be useful prognostic biomarkers for risk stratification of patients (29, 30).

The miRNAs utilized in the machine learning models presented in this study were chosen based on prior reporting suggesting that these miRNAs could distinguish ovarian cancer cases from controls (9). The miRNAs utilized in this model have been associated with many different functions. miR-150 has been associated with promoting ovarian cancer cell motility as well as enhancing apoptotic and anti-tumor effects of paclitaxel (31, 32). miR-200a has been shown in recent work to promote malignant behaviors through regulation of PCDH9 as well as invasion and metastasis *via* the ZEB axis (33, 34). Additional work has suggested this miRNA may promote cell invasion and migration through the PTEN pathway (35). A key feature of the neural network approach is that it can account for miRNAs that are either downregulated or upregulated. For example, miR-200c appears to be associated with anti-tumor properties (36). miR-203a has been shown to hinder the proliferation, migration and invasion of ovarian cancer cells through modulation of the AkT/GSK-3β/Snail signaling pathway (37). miR-335 has been studied both as a prognostic marker and in association with cisplatin sensitivity with several studies suggesting that this miRNA may be associated with a favorable prognosis through its actions within the BCL2 pathway (38–41). Utilizing the neural network approach allows a more comprehensive summary of various biologic processes which together may collectively point towards tumor growth.

The current study builds upon the prior report by showing that the serum miRNAs previously used to develop a general ovarian cancer diagnostic classifier can also be used to detect low-volume disease, potentially below the threshold of imaging. This may flag patients earlier in their disease course with asymptomatic, lower-volume disease where primary cytoreduction is closely linked to improved survival (42). Another potential application of this serum miRNA signature could be to apply it as a diagnostic tool for recurrences, although we did not test that possibility. Currently ovarian cancer recurrences are identified by rising CA-125 and or imaging such as computed tomography or PET scan and can be confirmed with biopsy as needed. A serum miRNA signature might allow for a non-invasive, “liquid biopsy” test to confirm recurrence without radiation or invasive biopsy.

We believe our study has several unique strengths. The neural network was verified with an independent cell line not used in the model creation and was tested over a range of tumor inoculums. In the clinical dataset, we tested our model specifically with early-stage patients, which are usually poorly represented in screening studies. We did not restrict our clinical dataset to only serous histology patients but rather reflected the heterogeneity of early-stage ovarian cancers seen in clinical practice. Even so, high grade histologies were well-represented. Moreover, we showed that we can distinguish ovarian cancers specifically from their benign histologic counterparts, which is a higher bar of stringency than if we had included other types of benign ovarian masses, such as simple cysts, benign teratomas, or fibromas, which are easily classified as benign based on sonographic imaging.

This study has several limitations as well. The cell line models used to create the xenograft neural network all had homologous repair defects (43). It is possible that BRCA-mutated ovarian tumors have a distinct miRNA profile as compared to BRCA wild-type cells and therefore this work may not be as applicable to women with BRCA wild-type tumors. However, the validation cell line OVCAR8 does not have a *BRCA* mutation, which implies that the neural network’s applicability is not restricted to *BRCA* mutated tumors. Moreover, among the patient samples we were agnostic to mutation status in most cases, and the model performed similarly. Similarly, the patient dataset included both tumors with favorable prognoses, such as Stage I, Grade 1 endometrioid adenocarcinomas, as well as tumors with relatively poorer prognoses, such as Stage II, Grade 3 serous adenocarcinomas. While one might argue that a tumor signature should focus only on aggressive histologies, a model which excludes other histologies risks providing false reassurance to patients and causing these highly curable tumors to be diagnosed at later stage, when the prognosis is similarly poor (44). Finally, the utility of the current model appears to be limited to high-risk populations, defined as those with an ovarian cancer prevalence of at least 1%. This is notably much lower than the prevalence of ovarian cancer in the general population, which is 0.07%. Clearly, improvements to the model would be needed to move towards general population screening. However, as no screening tools currently exist even for *BRCA1* mutation carriers, who have a 40% lifetime risk of ovarian cancer, we think that developing a screening tool with high sensitivity and negative predictive value in high-risk populations alone would already be a significant step forward.

In conclusion, a neural network model derived from miRNA serum signatures can identify either low volume or early-stage tumors. Whether the model can identify tumors that are both low volume and early stage will require larger human studies of patients with low volume, early-stage disease. In future studies, we hope to consider how a miRNA serum signature may be useful as an adjunct to other modalities to develop reliable screening for women at high risk for ovarian cancer.

## Data Availability Statement

The datasets presented in this study can be found in online repositories. The names of the repository/repositories and accession number(s) can be found in the article/**Supplementary Material**.

## Ethics Statement

The studies involving human participants were reviewed and approved by the Pelvic Mass Protocol (Brigham and Women’s Hospital Institutional Review Board Protocol 2000-P-001678) and the New England Case Control Study (Dana-Farber Cancer Institute Institutional Review Board Protocol 05–060). The patients/participants provided their written informed consent to participate in this study. The animal study was reviewed and approved by Dana-Farber Cancer Center Animal Resource Facility Ethics Guidelines (IACUC protocol 13-043).

## Author Contributions

(AG), Investigation, data collection, writing original draft; (SF), data collection, investigation; (KH), investigation; (NH), data collection, animal management; (WF), data curation, formal analysis, writing; (KS), data preprocessing, model validation, software; (KP), statistical analysis of human samples; (DWC), samples, writing; (DC), resources, conceptualization; (KE), overall PI, project conceptualization, formal analysis, Supervision, writing, and editing.

## Funding

The authors wish to acknowledge funding support from the Robert and Deborah First Family Fund (KME, DC), the Minnesota Ovarian Cancer Alliance (KME), the Mighty Moose Foundation (KME, DC), the Saltonstall Research Fund (KME, RSB), Potter Research Fund (KME, RSB), the Sperling Family Fund Fellowship (KME, RSB), the Bach Underwood Fund (KME, RSB), the White Foundation (AG, SJF), the First TEAM grant of the Foundation for Polish Science and the Smart Growth Operational Programme of the European Union (WF, KS, KP), NIH P50CA105009 (DWC, AFV), the William M Wood Foundation (DC) and the Honorable Tina Brozman Foundation (KME, DC).

## Conflict of Interest

The authors declare that the research was conducted in the absence of any commercial or financial relationships that could be construed as a potential conflict of interest.

## Publisher’s Note

All claims expressed in this article are solely those of the authors and do not necessarily represent those of their affiliated organizations, or those of the publisher, the editors and the reviewers. Any product that may be evaluated in this article, or claim that may be made by its manufacturer, is not guaranteed or endorsed by the publisher.

## Supplementary Material

Supplementary Data Sheet 1 miRNA mouse experiment data using the Fireplex platform.

Supplementary Data Sheet 2 miRNA expression in human serum samples. Values are log10-normalized.

Supplementary Data Sheet 3 NewNetXenograft-1.xml 1: Neural network trained on sera from three ovarian cancer xenografts using 14 miRNAs. 7miRTrimmedXenoNet-38.xml: Empirically optimizedneuralnetworkon sera fromthreeovarian cancer xenografts using7miRNAs. Signature-best-10.xml: Neural network trained on early-stage cohort samples.
